# Coronavirus rotational diffusivity

**DOI:** 10.1063/5.0031875

**Published:** 2020-11-01

**Authors:** M. A. Kanso, J. H. Piette, J. A. Hanna, A. J. Giacomin

**Affiliations:** 1 Chemical Engineering Department, Polymers Research Group, Kingston, Ontario K7L 3N6, Canada; 2 Mechanical Engineering Department, University of Nevada, Reno, Nevada 89557-0312, USA; 3 Physics, Engineering Physics and Astronomy Department, Queen’s University, Kingston, Ontario K7L 3N6, Canada; 4 Mechanical and Materials Engineering Department, Kingston, Ontario K7L 3N6, Canada

## Abstract

Just 11 weeks after the confirmation of first infection, one team had already discovered and published [D. Wrapp *et al.*, “Cryo-EM structure of the 2019-nCoV spike in the prefusion conformation,” Science **367**(6483), 1260–1263 (2020)] in exquisite detail about the new coronavirus, along with how it differs from previous viruses. We call the virus particle causing the COVID-19 disease *SARS-CoV-2*, a spherical capsid covered with spikes termed *peplomers*. Since the virus is not motile, it relies on its own random thermal motion, specifically the rotational component of this thermal motion, to align its peplomers with targets. The governing transport property for the virus to attack successfully is thus the rotational diffusivity. Too little rotational diffusivity and too few alignments are produced to properly infect. Too much, and the alignment intervals will be too short to properly infect, and the peplomer is wasted. In this paper, we calculate the rotational diffusivity along with the complex viscosity of four classes of virus particles of ascending geometric complexity: tobacco mosaic, gemini, adeno, and corona. The gemini and adeno viruses share icosahedral bead arrangements, and for the corona virus, we use polyhedral solutions to the Thomson problem to arrange its peplomers. We employ general rigid bead–rod theory to calculate complex viscosities and rotational diffusivities, from first principles, of the virus suspensions. We find that our *ab initio* calculations agree with the observed complex viscosity of the tobacco mosaic virus suspension. From our analysis of the gemini virus suspension, we learn that the fine detail of the virus structure governs its rotational diffusivity. We find the characteristic time for the adenovirus from general rigid bead–rod theory. Finally, from our analysis of the coronavirus suspension, we learn that its rotational diffusivity descends monotonically with its number of peplomers.

## I. INTRODUCTION

Shortly after the confirmation of first infection, one team had already discovered and published^1^ in exquisite detail about the new coronavirus, along with how it differs from previous viruses. We call the virus particle causing the COVID-19 disease *SARS-CoV-2*, a spherical capsid covered with hollow spikes termed *peplomers*. Since the virus is not motile, it relies on its own random thermal motion, specifically, the rotational component of this thermal motion, to align its peplomers with targets. From Fig. 1(B) of Ref. 2, we learn that to perfuse capsid contents into a cell, precisely two adjacent peplomers must align with a dimeric target, nominally rectangular (110 × 160 Å^2^). Furthermore, this alignment must be long enough for fusion. Once fused, perfusion progresses to infection.

Whereas much of the prior work on flow and the virus focuses on infection of the organism,^3–21^ this work targets the transport properties of the coronavirus particle and their implications in transport phenomena of cellular infection. Although our work is mainly driven by curiosity, it may deepen our understanding or even accelerate drug treatment or vaccine development, especially where these interfere with cellular infection.

The governing transport property for the virus to attack successfully is the rotational diffusivity of the SARS-CoV-2 particle (see Footnote 2 in p. 62 of Ref. 22). Too much rotational diffusivity and the alignment intervals will be too short to properly infect, and the peplomer is wasted. Too little rotational diffusivity, and too few alignments are produced to properly infect. The rotational diffusivity of a particle depends intimately on its shape.

Whereas in engineering, the complex viscosity function has a broad diversity of applications including polymer or suspension processing, for virus suspensions, its main use is for determining rotational diffusivity. In this paper, we calculate the complex viscosity and thus the rotational diffusivity of four classes of virus particles of ascending geometric complexity: tobacco mosaic, gemini, adeno, and corona. The gemini^23^ and adeno^24^ viruses share icosahedral bead arrangements, and for the corona virus, we use polyhedral solutions to the Thomson problem to arrange its peplomers.^25,26^ We employ general rigid bead–rod theory to calculate complex viscosities and rotational diffusivities, from first principles, of the virus suspensions (Refs. 27–34; see EXAMPLE 16.7-1 of Ref. 22 or EXAMPLE 13.6-1 of Ref. 35).

We are attracted to general rigid bead–rod theory first for its flexibility. We design each macromolecular structure, here virus particles, by rigidly connecting nearest bead centers with massless dimensionless rods. We are attracted to general rigid bead–rod theory second for the accuracy of its simplest special cases, the rigid dumbbell suspensions, for which many transport properties are predicted (see this reviewed in Sec. I of Ref. 31).

General rigid bead–rod theory proceeds from the continuity equation for the macromolecular configuration, called the *diffusion equation* [Eq. (13.2-13) of Ref. 22]. By *continuity*, we mean that the diffusion equation conserves orientation, preserving one and only one orientation per macromolecule. Hassager solves the diffusion equation for the configuration distribution function in small-amplitude oscillatory flows, which, for rigid macromolecules, reduces to the orientation distribution function, *ψ*(*θ*, *ϕ*, *t*).

Consider, for instance, a coronavirus particle close enough to fuse with a dimeric receptor.^2^ We refer the coronavirus particle to spherical coordinates and consider the receptor target orthogonal to its equator (*θ* = *π*/2) with the long axis of the dimeric receptor along the longitudinal direction. For this special infection opportunity, the probability of finding a peplomer aligned with said receptor is given by [see Eq. (9) of Ref. 36]

$$p\equiv \overset{\phi_{r}}{\underset{-\phi_{r}}{\int }}\overset{\theta_{r}}{\underset{-\theta_{r}}{\int }}\psi \left(\theta ,\phi ,t\right)\sin\theta d\theta d\phi ,$$ (1)

where for the nominally rectangular binding target,

$$\frac{\theta_{r}}{\phi_{r}}=\frac{110\text{ Å}}{160\text{ Å}}=\frac{11}{16}.$$ (2)

For fusion, we, of course, require two peplomers with said alignment,^2^ and thus, the probability of finding this falls well below *p*.

General rigid bead–rod theory connects *ψ* with macromolecular shapes, including those of viruses. In this way, the virus shape confers the transport properties to its suspension, including viscosity, elasticity, and diffusivities, be they rotational or translational. Little is known experimentally about the diffusivity of viruses, especially the rotational diffusivity. For instance, the translational diffusivity of the adenovirus has been measured by photon-correlation spectroscopy.^37^ The rotational diffusivity of tobacco mosaic viruses has also been measured by light scattering,^38–41^ transient electric birefringence,^42^ and flow birefringence.^43^ The rotational diffusivity is deducible from the translational one by the identity given in Sec. II below.

One of the challenges of *ab initio* calculations from general rigid bead–rod theory on coronaviruses is that the peplomer arrangement is not known. However, we do know that the spikes are charge-rich,^44,45^ and we can presume, charged identically. Furthermore, we know that the coronavirus spikes are anchored into its viral membrane and not into its capsid (Sec. 1. of Ref. 46), unlike the adenovirus spikes. Hence, the coronavirus spikes are free to be rearranged by their electrostatic repulsions. We thus expect the peplomers to arrange themselves by repelling one another into the polyhedral solutions to the Thomson problem.^25,26^ By the *Thomson problem*, we mean how identically charged particles will organize themselves onto a sphere by minimizing system potential energy. In this work, we are thus using minimum potential energy peplomer arrangements for our coronavirus model particles.

The rotational alignment of the virus particle studied herein is prefusion and not to be confused with the postfusion *diffusive rotational search* of the spike-protein unfolding that accompanies binding.^47^

## II. METHOD

Using general rigid bead–rod theory, we propose the construction of virus particles from sets of beads whose positions are fixed relative to one another. For example, the SARS-CoV-2 particle geometry is a spherical capsid surrounded by a constellation of protruding peplomers. We understand that the number of peplomers per virus particle differs from particle to particle and seems to decrease with time after inoculation. We suspend our bead–rod models of virus particles into a Newtonian solvent. We begin by neglecting interactions of the solvent velocity fields, be they (i) between nearest beads within the virus particle^48,49^ or (ii) between nearest virus particles. To any such collection of bead masses, we can associate a *moment of inertia ellipsoid* (MIE) whose center is the center of mass and whose principal moments of inertia match those of the virus particle. The MIE thus determines the orientability of the virus particle and thus the virus rotational diffusivity. Our use of moment of inertia ellipsoids is not to be confused with replacing the virus particle with an ellipsoid of revolution, with its own hydrodynamic environment.^50^ We know of no previous calculation of the moments of inertia ellipsoid of virus particles, and we think that this missing physics can deepen our understanding of SARS-CoV-2.

To model the virus particle, we locate each bead of mass ***m***_*i*_ with the position vector of the *i*th bead **r**_*i*_, where the virus particle center of mass **R** satisfies

$$\overset{N}{\underset{i=1}{\sum }}m_{i}\left(r_{i}-R\right)=0$$ (3)

so that

$$R=\frac{1}{M}\overset{N}{\underset{i=1}{\sum }}m_{i}r_{i},$$ (4)

where *N* is the total number of beads and $M\equiv \sum_{i=1}^{N}m_{i}$ is the virus particle mass. Since we construct our virus particles with identical beads of diameter *d* and mass *m*, then *M*…*mN*, and thus, the center of mass is

$$R=\frac{1}{N}\overset{N}{\underset{i=1}{\sum }}r_{i},$$ (5)

which we will use below.

We next install *viral coordinates* at the center of mass of the virus, and we orient these Cartesian coordinates such that ${\hat{\delta }}_{3}$ is along the polar axis of the *moment of inertia ellipsoid*. For our virus particles, ${\hat{\delta }}_{3}$ is through the particle. In this study, to allow us to explore the surface density of peplomers, the peplomer arrangement, and even the triadic details of the three-glycoprotein spikes, we will use a finely beaded sphere for the capsid. By necessity of general rigid bead–rod theory, our capsid and peplomer beading must be equally fine.

The position vector of the *i*th bead with respect to the virus center of mass is given by

$$R_{i}\equiv \left[R_{i1},R_{i2},R_{i3}\right].$$ (6)

We define the principal moments of inertia *I*_1_, *I*_2_, and *I*_3_ by [Eqs. (16.7-17) and (16.7-18) of Ref. 22 or (13.6-17) and (13.6-18) of Ref. 35]

$$I_{1}\equiv m\overset{N}{\underset{i=1}{\sum }}\left(R_{i2}^{2}+R_{i3}^{2}\right),$$ (7)

$$I_{2}\equiv m\overset{N}{\underset{i=1}{\sum }}\left(R_{i1}^{2}+R_{i3}^{2}\right),$$ (8)

$$I_{3}\equiv 2m\overset{N}{\underset{i=1}{\sum }}R_{i1}^{2},$$ (9)

where the subscript *i* is the bead number. We design each virus particle structure by first rigidly connecting nearest bead centers with massless widthless rods. Throughout our work, *L* is the distance between the nearest bead centers. We then complete the general rigid bead–rod construction by rigidly connecting the remaining bead centers to their nearest neighbors. For the SARS-CoV-2 particle, *L* is the center to center distance between osculating beads forming the capsid. Although the peplomer is a spike with a bulbous triadic head, in this work, we will model it as a single bead not touching the capsid.

Since the virus particle structure is axisymmetric, so will be its moment of inertia ellipsoid. By *axisymmetric*, we mean that both the virus particle and its moment of inertia ellipsoid have at least one axis of symmetry.^27^ Furthermore, if the virus particle structure is axisymmetric, at least two of its principal moments of inertia equate, at any angle from the molecular axis, so that *I*_1_ = *I*_2_. Our usage of *axisymmetric* is not to be confused with the common geometric meaning of continuous rotational symmetry about an axis.

Hassager derives the expression for the dimensionless shear relaxation function for general rigid bead–rod theory,

$$\frac{G\left(s\right)}{nkT}=\frac{\delta \left(s\right)}{kT}\left(\frac{2\eta_{s}}{n}+\zeta L^{2}a\right)+be^{-s/\lambda },$$ (10)

in which [Eq. (16.7-38) of Ref. 22 or Eqs. (13.6-44), (13.6-45), and (13.6-46) of Ref. 35]

$$a\equiv \frac{2I_{1}+I_{3}}{6mL^{2}}-\frac{{\left(I_{1}-I_{3}\right)}^{2}}{5mL^{2}I_{1}},$$ (11)

$$b\equiv \frac{3{\left(I_{1}-I_{3}\right)}^{2}}{5I_{1}^{2}}$$ (12)

and the particle rotation constant is^29,31^

$$\nu \equiv \frac{6mL^{2}}{I_{1}},$$ (13)

where 0 ≤ *b* ≤ 3/5 and 0 ≤ *aν* ≤ 7/2. The three quantities *a*, *b*, and *ν* thus define completely the differences in linear viscoelastic behaviors arising between different axisymmetric macromolecular structures. Whereas we associate *a* with the Dirac delta function contribution to the relaxation function, we associate *b* with the dying exponential.

The relaxation time of the corresponding virus particle suspension can be expressed as

$$\lambda \equiv \frac{\zeta I_{1}}{6mkT}\equiv \frac{\zeta L^{2}}{\nu kT}$$ (14)

in which the bead friction coefficient is given by

$$\zeta \equiv 3\pi d\eta_{s}.$$ (15)

We define a characteristic time for all virus particle suspensions as

$$\lambda_{0}\equiv \frac{\zeta L^{2}}{12kT}=\frac{\pi d\eta_{s}L^{2}}{4kT},$$ (16)

which nondimensionalizes as

$$\frac{nkT\lambda_{0}}{\eta_{s}}\equiv \frac{3}{4}\varphi {\left(\frac{L}{d}\right)}^{2},$$ (17)

where *φ* is the bead volume fraction, and for osculating beads, where *L* = *d*,

$$\lambda_{0}=\frac{\pi d^{3}\eta_{s}}{4kT}$$ (18)

and

$$\frac{nkT\lambda_{0}}{\eta_{s}}\equiv \frac{3}{4}\varphi .$$ (19)

Dividing Eq. (14) by Eq. (16) normalizes the relaxation time,

$$\frac{\lambda }{\lambda_{0}}\equiv \frac{12}{\nu }.$$ (20)

We can then use Eq. (10) to calculate the polymer contribution to the stress tensor in any linear viscoelastic flow, including oscillatory shear flow, from [Eq. (1) of Ref. 30]

$$\tau_{p}\equiv -\int_{-\infty }^{t}G\left(t-t^{\prime }\right)\dot{\gamma }\left(t^{\prime }\right)dt^{\prime },$$ (21)

where all symbols are defined in Tables I and II.

**TABLE I.** Dimensional variables *M* ≡ mass, *L* ≡ length, and *t* ≡ time.

Name	Unit	Symbol
Angular frequency	*t*^−1^	*ω*
Avogadro constant	mol^−1^	Ñ
Bead diameter	*L*	*d*
Bead friction coefficient [Eq. (15)]	*M*/*t*	*ζ*
Capsid radius (see Figs. 4 and 8)	*L*	*r*_*c*_
Cartesian coordinates	*L*	*x*, *y*, *z*
Cartesian coordinates with respect to the center of mass	*L*	${\hat{\delta }}_{1},{\hat{\delta }}_{2},{\hat{\delta }}_{3}$
Characteristic time for each virus particle suspension	*s*	*λ*_*s*_
Characteristic time, zero-shear	*t*	*λ*_*c*_
Complex viscosity [Eq. (36)]	*M*/*Lt*	η^*^
Density	*M*/*L*^3^	*ρ*
Edge vector pointing from adenovirus vertex *i* to vertex *j*	*L*	**E**_*ij*_
Element for Kronecker delta [Eq. (10)]	*t*^−1^	*δ*(*s*)
Energy values in molecular-scale systems	*ML*^2^/*t*^2^	*kT*
Intrinsic minus imaginary part of non-linear complex viscosity	*L*^3^/*M*	[*η*″]
Intrinsic real part of non-linear complex viscosity	*L*^3^/*M*	[*η*′]
Intrinsic zero-shear viscosity	*L*^3^/*M*	[*η*]_0_
Macromolecular center of mass [Eq. (5)]	*L*	**R**
Mass concentration	*M*/*L*^3^	*c*
Mass of each bead	*M*	*m*_*i*_
Minus imaginary part of non-linear complex viscosity [Eq. (35)]	*M*/*Lt*	*η*″
Moments of inertia [Eqs. (7)–(9)]	*ML*^2^	*I*_1_, *I*_2_, *I*_3_
Number of dumbbells per unit volume	1/*L*^3^	*n*
Peplomer bulb center radial position (see Fig. 8)	*L*	*r*_*p*_ ≡ *r*_v_ − *r*_*b*_
Peplomer bulb radius (see Fig. 8)	*L*	*r*_*b*_
Polymer contribution to the stress tensor [Eqs. (21) and (33)]	*M*/*Lt*^2^	*τ*_*p*_
Position vector of the *ith* bead and *jth* element with respect to the center of mass [Eq. (6)]	*L*	*R*_*ij*_
Position vector of the *ith* bead with respect to the center of mass [Eq. (6)]	*L*	**R**_*i*_
Position vector of the *ith* bead [Eq. (5)]	*L*	**r**_*i*_
Position vector of adenovirus vertex *i* with respect to the center of mass	*L*	**V**_*i*_
Real part of non-linear complex viscosity [Eq. (34)]	*M*/*Lt*	*η*′
Reduced angular frequency	*M*/*L*^3^	*ω*_*R*_
Relaxation time of rigid dumbbell [Eq. (16)]	*t*	*λ*_0_
Relaxation time of solution Eq. (14)	*t*	*λ*
Rotational diffusivity	*s*^−1^	*D*_*r*_
Rotatory diffusivity	*L*^2^/*t*	*D*_rot_
Shear rate amplitude [Eq. (29)]	*t*^−1^	${\dot{\gamma }}^{0}$
Shear rate at specific time *t*^′^ [Eq. (21)]	*t*^−1^	$\dot{\gamma }$(*t*′)
Shear rate tensor [Eq. (29)]	*t*^−1^	$\dot{\gamma }\left(t\right)$
Shear rate [Eq. (29)]	*t*^−1^	$\dot{\gamma }\left(t\right)$
Shear relaxation function [Eq. (10)]	*M*/*Lt*^2^	*G*(*s*)
Solvent viscosity	*M*/*Lt*	*η*_*s*_
Specific time [Eq. (21)]	*t*	*t*′
Temperature	*T*	*T*
Time	*t*	*t*
Time difference	*t*	*s* ≡ *t* − *t*′
Total mass	*M*	*M*
Translational diffusivity	*L*^2^/*t*	*D*_*tr*_
Virus radius (see Figs. 4 and 8)	*L*	*r*_v_ ≡ *r*_*p*_ + *r*_*b*_
Viscosity, zero-shear	*M*/*Lt*	*η*_0_
Zero-shear first normal stress difference	*M*/*L*	*Ψ*_0,1_

**TABLE II.** Dimensionless variables and groups.

Name	Symbol
Bead volume fraction [Eq. (17)]	*φ*
Coefficient in Eq. (11)	*a*
Coefficient in Eq. (12)	*b*
Coefficient in Eq. (13)	*ν*
Deborah number, oscillatory shear	De ≡ *λω*
Golden Ratio	*β*
Relaxation time ratio	Λ ≡ *λ*/*λ*_0_
Total number of beads	*N*
Total number of peplomers	*N*_*p*_
Total number of capsid beads	*N*_*c*_
Weissenberg number	$\text{Wi}\equiv \lambda {\dot{\gamma }}^{0}$
Probability	*p*
Orientation distribution	*ψ*(*θ*, *ϕ*, *t*)
Spherical coordinate, latitudinal	*θ*
Spherical coordinate, longitudinal	*ϕ*
Spherical coordinate, latitudinal receptor	*θ*_*r*_
Spherical coordinate, longtitudinal receptor	*ϕ*_*r*_

In this work, we apply these derivations to virus particles, specifically to the calculation of the rotational diffusivity, given by the identity (see Footnote 2 of p. 62 of Ref. 22)

$$D_{r}\equiv \frac{1}{6\lambda }$$ (22)

about which, for virus particles, remarkably little is known. Substituting Eq. (20) into Eq. (22) and nondimensionalizing,

$$\lambda_{0}D_{r}=\frac{\nu }{72}.$$ (23)

Substituting Eq. (17) into this and rearranging gives the *dimensionless rotational diffusivity*

$$\lambda_{s}D_{r}\equiv \frac{\eta_{s}}{nkT}D_{r}=\frac{\nu }{54\varphi }{\left(\frac{d}{L}\right)}^{2}$$ (24)

from which we uncover a characteristic time for each virus particle suspension *λ*_*s*_. The quantity *ν* thus defines completely the rotational diffusivity of a virus particle.

In the tradition of the transport sciences, we define the *rotatory diffusivity* as (see Footnote 2 of p. 62 of Ref. 22)

$$D_{\text{rot}}\equiv \frac{2kT}{\zeta },$$ (25)

which, for any axisymmetric macromolecule, from general rigid bead–rod theory, gives

$$D_{\text{rot}}\equiv \frac{12L^{2}}{\nu }D_{r},$$ (26)

which has the dimensions of diffusivity and which is four times the *translational diffusivity*,

$$D_{\text{rot}}\equiv 4D_{tr}$$ (27)

or

$$D_{r}\equiv \frac{\nu }{3L^{2}}D_{tr}.$$ (28)

In this paper, we depart from said transport tradition of using the rotatory diffusivity, *D*_rot_, and frame our results in terms of the rotational diffusivity, *D*_*r*_.

The challenge in determining the rotational diffusivity of a virus particle, from first principles, begins with modeling its intricate geometry with beads, locating the position of each bead. Once overcome, the next challenge is to use this geometry to arrive at the transport properties for the SARS-CoV-2 particle. From these, we will deepen our understanding of how these remarkable particles can align their peplomers both for long enough and often enough to infect.

**FIG. 1.**
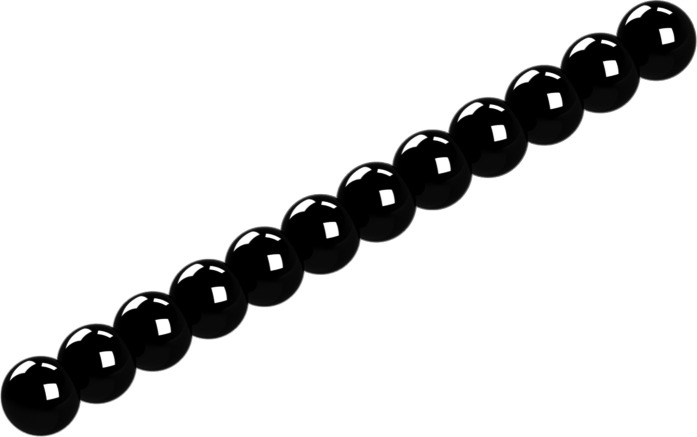
General rigid bead–rod model of tobacco mosaic virus, *N* = 12.

**FIG. 2.**
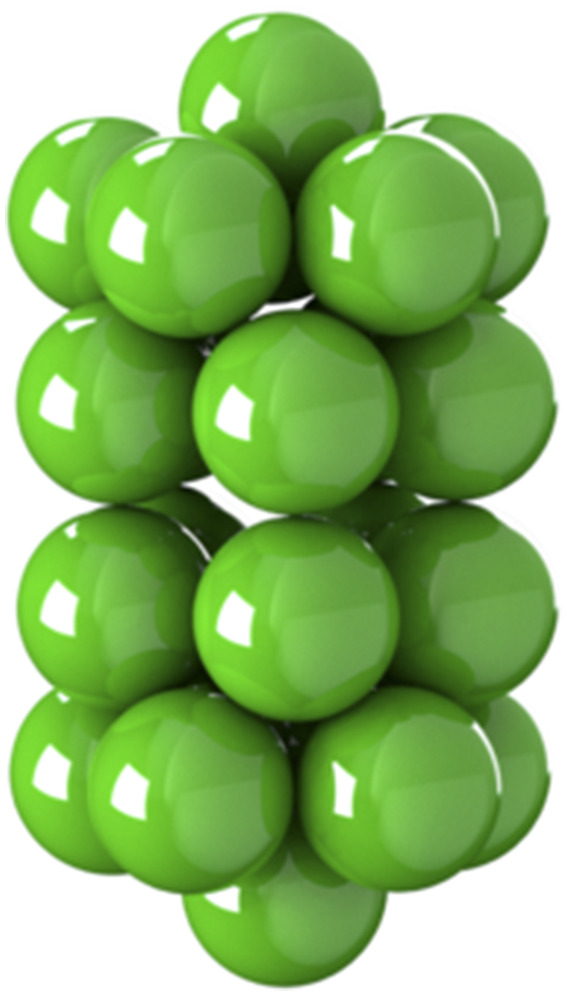
General rigid bead–rod model of gemini virus, *N* = 22. See Fig. 2 of Ref. 23.

**FIG. 3.**
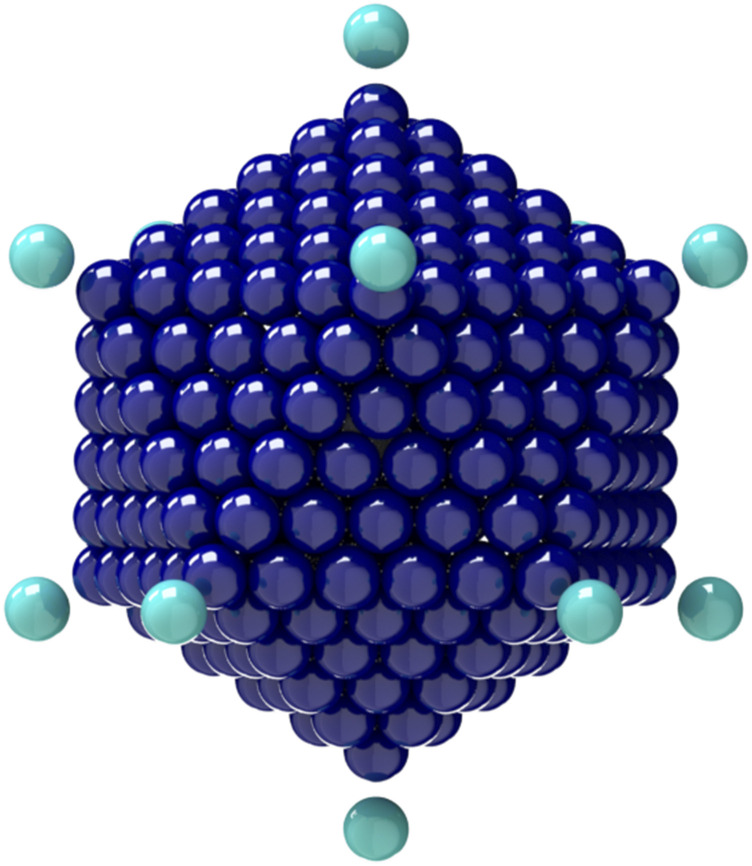
General rigid bead–rod model of adenovirus, *N*_*c*_ = 252, *N*_*p*_ = 12, and *r*_v_/*r*_*c*_ = 5/4.

**FIG. 4.**
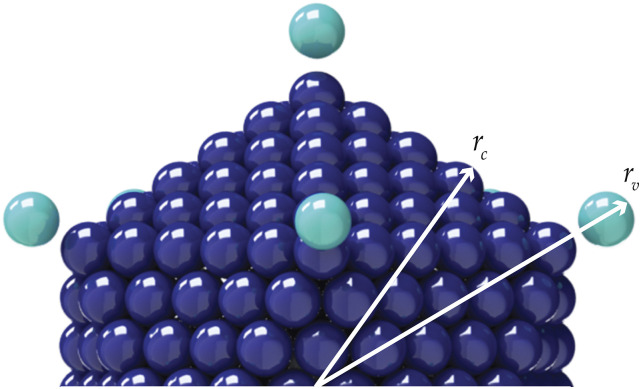
Connections between adenovirus particle dimensions and its general rigid bead–rod model.

**FIG. 5.**
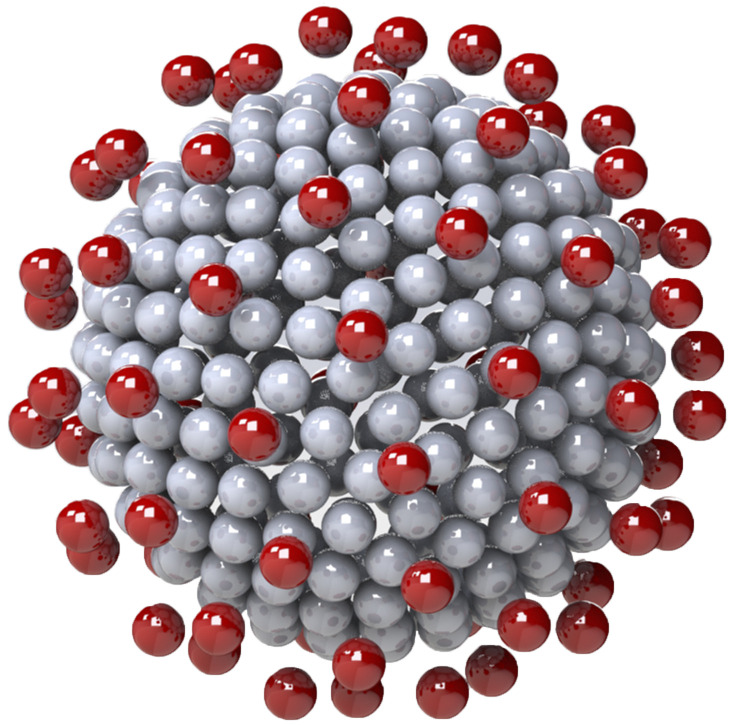
General rigid bead–rod model of coronavirus, *N*_*c*_ = 256, *N*_*p*_ = 74, and *r*_v_/*r*_*c*_ = 5/4.

For this work, we chose general rigid bead–rod theory for its flexibility and accuracy (Sec. I of Ref. 31). However, for bead–rod structures as complex as coronaviruses, drawing the bead–rod models presented a challenge, which we met using solid modeling computer-aided design.^51^ This challenge arises when progressing from the **R** values in Eq. (5) to bead–rod imagery, for instance, when going from Table III for the **R** values of our tobacco mosaic and gemini viruses to our images in Figs. 1–5, respectively.

**TABLE III.** Bead positions for tobacco mosaic and gemini viruses.

Macromolecule	*L*[*R*_*i*1_, *R*_*i*2_, *R*_*i*3_]
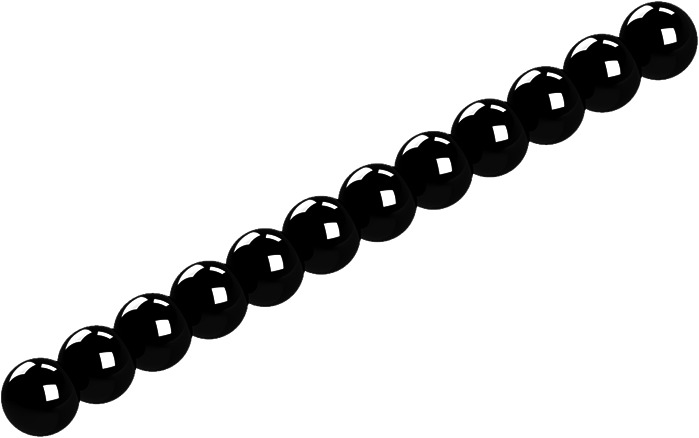	$\begin{array}{c} \left[0,0,-\frac{11}{2}\right];\left[0,0,-\frac{9}{2}\right];\left[0,0,-\frac{7}{2}\right];\left[0,0,-\frac{5}{2}\right];\left[0,0,-\frac{3}{2}\right];\left[0,0,-\frac{1}{2}\right];\\ \left[0,0,\frac{11}{2}\right];\left[0,0,\frac{9}{2}\right];\left[0,0,\frac{7}{2}\right];\left[0,0,\frac{5}{2}\right];\left[0,0,\frac{3}{2}\right];\left[0,0,\frac{1}{2}\right] \end{array}$
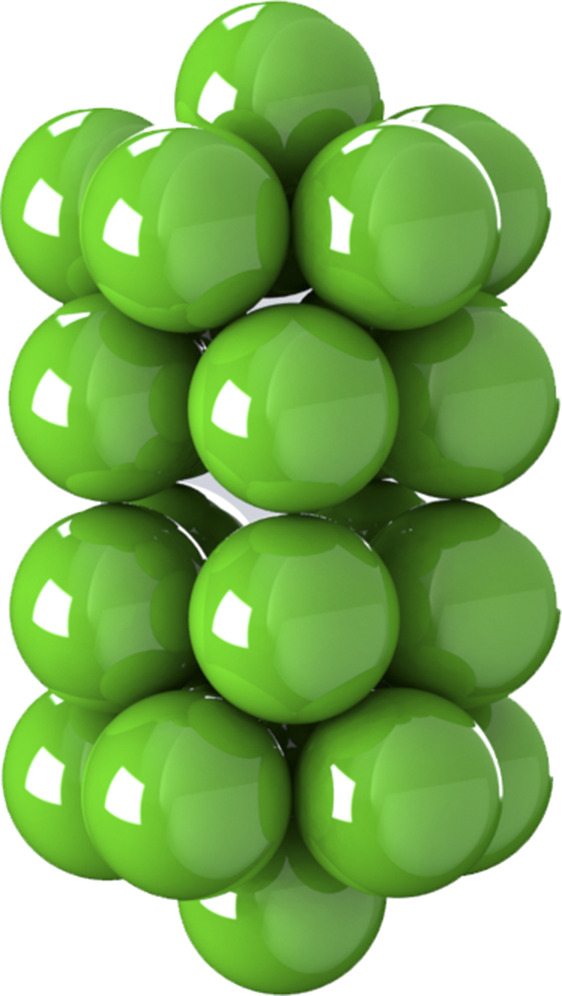	$\begin{array}{c} \left[0,0,x_{1}\right];\left[-x_{2},0,\frac{1}{2}\right];\left[x_{2},0,x_{3}\right];\left[x_{4},-\frac{1}{2},\frac{1}{2}\right];\\ \left[x_{4},\frac{1}{2},\frac{1}{2}\right];\left[-x_{4},-\frac{1}{2},x_{3}\right];\left[-x_{4},\frac{1}{2},x_{3}\right];\\ \left[-x_{5},-x_{6},\frac{1}{2}\right];\left[-x_{5},x_{6},\frac{1}{2}\right];\left[x_{5},-x_{6},x_{3}\right];\\ \left[x_{5},x_{6},x_{3}\right];\left[0,0,-x_{1}\right];\left[-x_{2},0,-\frac{1}{2}\right];\left[x_{2},0,-x_{3}\right];\\ \left[x_{4},-\frac{1}{2},-\frac{1}{2}\right];\left[x_{4},\frac{1}{2},-\frac{1}{2}\right];\left[-x_{4},-\frac{1}{2},-x_{3}\right];\\ \left[-x_{4},\frac{1}{2},-x_{3}\right];\left[-x_{5},-x_{6},-\frac{1}{2}\right];\left[-x_{5},x_{6},-\frac{1}{2}\right];\\ \left[x_{5},x_{6},-x_{3}\right];\left[x_{5},x_{6},-x_{3}\right]\\ \text{where}\\ x_{1}\equiv \frac{1}{2}+\frac{5}{\sqrt{50-10\sqrt{5}}}+\frac{1}{\sqrt{10-2\sqrt{5}}},x_{2}\equiv \sqrt{\frac{2}{5-\sqrt{5}}},\\ x_{3}\equiv \frac{1}{2}+\frac{2}{\sqrt{10-2\sqrt{5}}},x_{4}\equiv \frac{1}{2}\sqrt{\frac{5}{10-2\sqrt{5}}}+\frac{1}{2\sqrt{10-2\sqrt{5}}},\\ x_{5}\equiv \frac{1}{2\sqrt{10-2\sqrt{5}}}-\frac{1}{2}\sqrt{\frac{5}{10-2\sqrt{5}}},x_{6}\equiv \frac{1}{2}\sqrt{\frac{5+\sqrt{5}}{5-\sqrt{5}}} \end{array}$

In Secs. IV–VII, we calculate the rotational diffusivity along with the complex viscosity of four classes of virus particles of ascending geometric complexity: tobacco mosaic, gemini, adeno, and corona. Section IV affords a comparison of our general bead–rod theory with measured behavior of the complex viscosity. Section V is purposed to explore how fine structural detail affects virus rotational diffusivity. Section VI affords a comparison with the measured value of the translational diffusivity, and Sec. VII, affords an exploration of how the detailed structure of SARS-CoV-2 affects its rotational diffusivity.

## III. OSCILLATORY SHEAR FLOW

One measures the complex viscosity in oscillatory shear flow generated by confining the fluid to a simple shear apparatus and then by subjecting one solid–liquid boundary to a coplanar sinusoidal displacement, generating the corresponding cosinusoidal shear rate

$$\dot{\gamma }\left(t\right)={\dot{\gamma }}^{0}\cos\omega t$$ (29)

such that the rate of deformation tensor is given by

$$\dot{\gamma }\left(t\right)=\left[\begin{array}{ccc} 0 & {\dot{\gamma }}^{0}\cos\omega t & 0\\ {\dot{\gamma }}^{0}\cos\omega t & 0 & 0\\ 0 & 0 & 0 \end{array}\right].$$ (30)

Using the characteristic relaxation time of the virus suspension, *λ*, we can nondimensionalize Eq. (29) as

$$\lambda \dot{\gamma }\left(t\right)=\lambda {\dot{\gamma }}^{0}\cos\lambda \omega \left(t/\lambda \right),$$ (31)

where *λω* and $\lambda {\dot{\gamma }}^{0}$ are the Deborah and Weissenberg numbers. In this paper, we focus on small-amplitude oscillatory shear flow (SAOS). For this flow field, for the molecular definition of *small amplitude*, general rigid bead–rod theory yields

$$\lambda {\dot{\gamma }}^{0}\ll \frac{1}{\nu \sqrt{2}}$$ (32)

whose left side is the macromolecular Weissenberg number. From Eq. (32), we learn that structures with higher *ν* will have lower limits for linear viscoelasticity.

Substituting Eqs. (10) and (29) into Eq. (21) yields the polymer contribution to the shear stress

$$\tau_{p}={\dot{\gamma }}^{0}\left\{\left[\eta^{\prime }\left(\omega \right)-\eta_{s}\right]\cos\omega t+\eta^{\prime \prime }\left(\omega \right)\sin\omega t\right\}$$ (33)

in which [Eqs. (40) and (41) of Ref. 31]

$$\frac{\eta^{\prime }-\eta_{s}}{\eta_{0}-\eta_{s}}={\left(\frac{1}{2b/a\nu }+1\right)}^{-1}\left(\frac{1}{2b/a\nu }+\frac{1}{1+{\left(\lambda \omega \right)}^{2}}\right),$$ (34)

$$\frac{\eta^{\prime \prime }}{\eta_{0}-\eta_{s}}={\left(\frac{1}{2b/a\nu }+1\right)}^{-1}\frac{\lambda \omega }{1+{\left(\lambda \omega \right)}^{2}},$$ (35)

where

$$\eta^{*}\equiv \eta^{\prime }-i\eta^{\prime \prime }$$ (36)

is the complex viscosity.^52,53^ In this paper, we plot the real and imaginary parts of the responses as functions of frequency, following the work of Ferry (Secs. 2.A.4–2.A.6 of Ref. 54) or Bird *et al.* (Sec. 4.4 of Ref. 55).

As *ω* → 0, for the polymer contribution to the zero-shear viscosity, we get

$$\frac{\eta_{0}-\eta_{s}}{nkT\lambda }=\frac{a\nu }{2}+b=b\left[1+\frac{2b}{a\nu }\right]{\left(\frac{2b}{a\nu }\right)}^{-1},$$ (37)

which we use for Tables VI–IX.

Following EXAMPLE 5.2-6 of Ref. 55 and specifically by setting *n*′ = *n* = 1 in Eq. (5.2-4) of Ref. 55, we can define a structure-dependent characteristic time,

$$\lambda_{c}\equiv \frac{\Psi_{0,1}}{\eta_{0}-\eta_{s}},$$ (38)

which is the ratio of the first normal stress coefficient to the viscosity at zero shear rate and thus reflects fluid elasticity. We insert Eq. (44) of Ref. 31 to get the structure-dependent characteristic time

$$\lambda_{c}=2\lambda {\left(\frac{1}{2b/a\nu }+1\right)}^{-1}$$ (39)

into which we insert Eq. (20) to get

$$\frac{\lambda_{c}}{\lambda_{0}}=\frac{24}{\nu }{\left(\frac{1}{2b/a\nu }+1\right)}^{-1},$$ (40)

which we will use below.

## IV. TOBACCO MOSAIC

In this section, we test the use of general rigid bead–rod theory for predicting the complex viscosity of viruses by comparing with the measured values for tobacco mosaic virus suspensions. Although this particular virus has the form of a nanotube (see Fig. 1 of Ref. 24), since its bore is narrow, we shall approximate this rigid and rod-like virus with an osculated shish-kebab (see Table VI). From general rigid bead–rod theory we know that, for the osculated shish-kebab (TABLE XV of Ref. 31),

$$\frac{\lambda }{\lambda_{0}}=\frac{1}{6}N\left(N^{2}-1\right)$$ (41)

in which *λ*_0_ is given by Eq. (18) and *d* is the diameter of the tobacco mosaic virus (*d* ≃ 18 nm from Ref. 56).

We will next test Eqs. (34) and (35) against the well-known behaviors of the complex viscosities of the tobacco mosaic suspensions (see Ref. 57 and Fig. 9-3 of Ref. 54). Proceeding specifically from the data in Fig. 14.5-1 of Ref. 22 and mindful of [Eq. (14.4-23) of Ref. 22],

$$\lambda =\frac{\eta_{0}-\eta_{s}}{nkT}=\frac{{\left[\eta \right]}_{0}\eta_{s}M}{ÑkT}$$ (42)

so that

$$\lambda \omega =\frac{{\left[\eta \right]}_{0}\eta_{s}M}{ÑkT}\omega ={\left[\eta \right]}_{0}\omega_{R},$$ (43)

and mindful of Eqs. (4.4-16) and (4.4-17) of Ref. 55,

$${\left[\eta \right]}_{0}\equiv \underset{c\to 0}{\lim}\frac{\eta_{0}-\eta_{s}}{c\eta_{s}},$$ (44)

$$\left[\eta^{\prime }\right]\equiv \underset{c\to 0}{\lim}\frac{\eta^{\prime }-\eta_{s}}{c\eta_{s}},$$ (45)

$$\frac{\left[\eta^{\prime \prime }\right]}{\omega }\equiv \underset{c\to 0}{\lim}\frac{\eta^{\prime \prime }/\omega }{c\eta_{s}}$$ (46)

so that for dilute virus suspensions, where

$$\frac{c}{\rho }\ll 1,$$ (47)

we get

$${\left[\eta \right]}_{0}≃\frac{\eta_{0}-\eta_{s}}{c\eta_{s}},$$ (48)

$$\left[\eta^{\prime }\right]≃\frac{\eta^{\prime }-\eta_{s}}{c\eta_{s}},$$ (49)

$$\frac{\left[\eta^{\prime \prime }\right]}{\omega }≃\frac{\eta^{\prime \prime }/\omega }{c\eta_{s}}$$ (50)

so that

$$\frac{\left[\eta^{\prime }\right]}{{\left[\eta \right]}_{0}}≃\frac{\eta^{\prime }-\eta_{s}}{\eta_{0}-\eta_{s}},$$ (51)

$$\frac{\left[\eta^{\prime \prime }\right]}{{\left[\eta \right]}_{0}}≃\frac{\eta^{\prime \prime }}{\eta_{0}-\eta_{s}}.$$ (52)

**FIG. 6.**
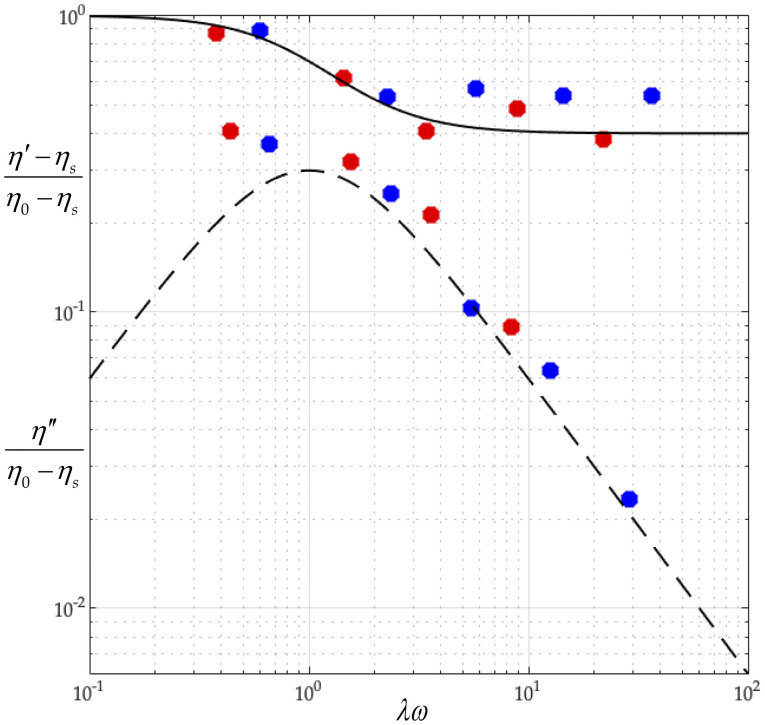
The dimensionless complex viscosity of tobacco mosaic virus suspension predicted by the general rigid bead–rod theory vs experimental data (Fig. 14.5-1 of Ref. 22). The data are for solutions of tobacco mosaic virus of *M* = 3.9 × 10^7^ g/mol. The red points represent data taken at 310 K, and the blue ones are taken at 298.2 K. The solvent viscosity at the two temperatures is *η*_*s*_ = 3.43 × 10^−3^ Pa s and *η*_*s*_ = 5.16 × 10^−3^ Pa s, respectively. The general rigid bead–rod theory predictions are computed using a value [*η*]_0_ = 18 cm^3^/g as the best fit with the data. The solid curve describes (*η*′ − *η*_*s*_)/(*η*_0_ − *η*_*s*_), and the dashed one describes *η*″/(*η*_0_ − *η*_*s*_).

We construct Fig. 6 using the best fit values of [*η*]_0_ = 23.35 and, at *T* = 310.0 K, *λ* = 5.20 × 10^−4^ s and, at *T* = 298.2 K, *λ* = 8.10 × 10^−4^ s. By Eq. (22), these correspond to rotational diffusivities at *T* = 310.0 K and *D*_*r*_ = 321 s^−1^ and at *T* = 298.2 K and *D*_*r*_ = 206 s^−1^.

In passing, mindful of the caption of Fig. 14.5-1 of Ref. 22, we calculate the dimensional relaxation times for the tobacco mosaic virus suspensions in Fig. 6 at *T* = 310.0 K,

$$\begin{array}{ccc} \lambda & = & \frac{{\left[\eta \right]}_{0}\eta_{s}M}{ÑkT}\\ & = & \frac{20\left(\text{c}{\text{m}}^{3}/\text{g}\right)3.43\times 10^{-3}\left(\text{Pa s}\right)3.9\times 10^{7}\left(\text{g}/\text{mol}\right)}{6.022\times 10^{23}\left(\text{mo}{\text{l}}^{-1}\right)1.380\times 10^{-23}\left(\text{J}/\text{K}\right)310.0\left(\text{K}\right)}\\ & = & 1.04\times 10^{-3}\text{ s,} \end{array}$$ (53)

and at *T* = 298.2 K,

$$\begin{array}{ccc} \lambda & = & \frac{{\left[\eta \right]}_{0}\eta_{s}M}{ÑkT}\\ & = & \frac{20\left(\text{c}{\text{m}}^{3}/\text{g}\right)5.16\times 10^{-3}\left(\text{Pa s}\right)3.9\times 10^{7}\left(\text{g}/\text{mol}\right)}{6.022\times 10^{23}\left(\text{mo}{\text{l}}^{-1}\right)1.380\times 10^{-23}\left(\text{J}/\text{K}\right)298.2\left(\text{K}\right)}\\ & = & 1.62\times 10^{-3}\text{ s}. \end{array}$$ (54)

The fitted value [*η*]_0_ = 23.35 falls below the reported theoretical value of [*η*]_0_ = 27 (see the caption of Fig. 14.5-1 of Ref. 22).

Solving Eq. (52) for the integer number of beads in the osculated shish-kebab,

$$N=\frac{{\left(\sqrt{243\Lambda^{2}-1}+\Lambda \sqrt{3^{5}}\right)}^{2/3}+1}{\sqrt{3}{\left(\sqrt{243\Lambda^{2}-1}+\Lambda \sqrt{3^{5}}\right)}^{1/3}},$$ (55)

where

$$\Lambda \equiv \frac{\lambda }{\lambda_{0}}$$ (56)

and where the right side of Eq. (55) is rounded to the nearest integer. Using Eq. (18) and for *d* ≃ 18 nm, at *T* = 310.0 K, we get

$$\lambda_{0}=\frac{\pi }{4}\frac{{\left(18\times 10^{-9}\right)}^{3}3.43\times 10^{-3}\left(\text{Pa s}\right)}{1.380\times 10^{-23}\left(\text{J}/\text{K}\right)310.0\left(\text{K}\right)}=3.67\times 10^{-6}\text{ s,}$$ (57)

and at *T* = 298.2 K,

$$\lambda_{0}=\frac{\pi }{4}\frac{{\left(18\times 10^{-9}\right)}^{3}5.16\times 10^{-3}\left(\text{Pa s}\right)}{1.380\times 10^{-23}\left(\text{J}/\text{K}\right)298.2\left(\text{K}\right)}=5.74\times 10^{-6}\text{ s.}$$ (58)

Thus, using Eq. (57) with the fitted value of *λ* = 5.20 × 10^−4^ s for *T* = 310.0 K gives Λ = 283, and so, Eq. (55) gives *N* = 12. Similarly, using that with *λ* = 8.10 × 10^−4^ s for *T* = 298.2 K gives Λ = 282, and so, *N* = 12. From this, we learn that the tobacco mosaic virus can be modeled with an osculated shish-kebab of 12 beads, for which Table III lists the position vectors (see Fig. 1).

Recall that, below Eq. (52), we found that at *T* = 310.0 K, *D*_*r*_ = 321 s^−1^ (or *λ*_0_*D*_*r*_ = 1.17 × 10^−3^), and at *T* = 298.2 K, *D*_*r*_ = 206 s^−1^ (or *λ*_0_*D*_*r*_ = 1.18 × 10^−3^). These values compare closely with the value predicted by Eq. (23) for an osculated shish-kebab of *N* = 12. Furthermore, from the available measurements (all non-rheological), the rotational diffusivity range for the tobacco mosaic virus at room temperature (20°C–25°C) is^38–43^

$$285\leq D_{r}\leq \;400\;{\text{s}}^{-1}.$$ (59)

Our value of *D*_*r*_ = 206 s^−1^, fitted to complex viscosity measurements (Fig. 6), falls just below this range.

In this section, we have approximated this rigid and rod-like virus with an osculated shish-kebab. However, its detailed structure of a narrow-bore nanotube consisting of the osculated helix of beads shown in Fig. 1. of Ref. 24 can be captured using Eqs. (5) and (6) of Ref. 28, where *L* = *d*. We leave this for another day.

## V. GEMINI

In this section, we use general rigid bead–rod theory to model the gemini virus as twin truncated icosahedra. To illustrate these twin truncated icosahedra, we construct Fig. 2 from the position vectors in Table III. We then compare this twin icosahedral structure to its coarser simpler cousin, two osculating beads (*L* = 2*R*). From Table VII, we learn that the finer twin icosahedral structure of the gemini virus increases both *λ*/*λ*_0_ and (*η*_0_ − *η*_*s*_)/*nkTλ* over two osculating beads. From this, we deepen our understanding of the role played by the finer twin truncated icosahedral structure.

**FIG. 7.**
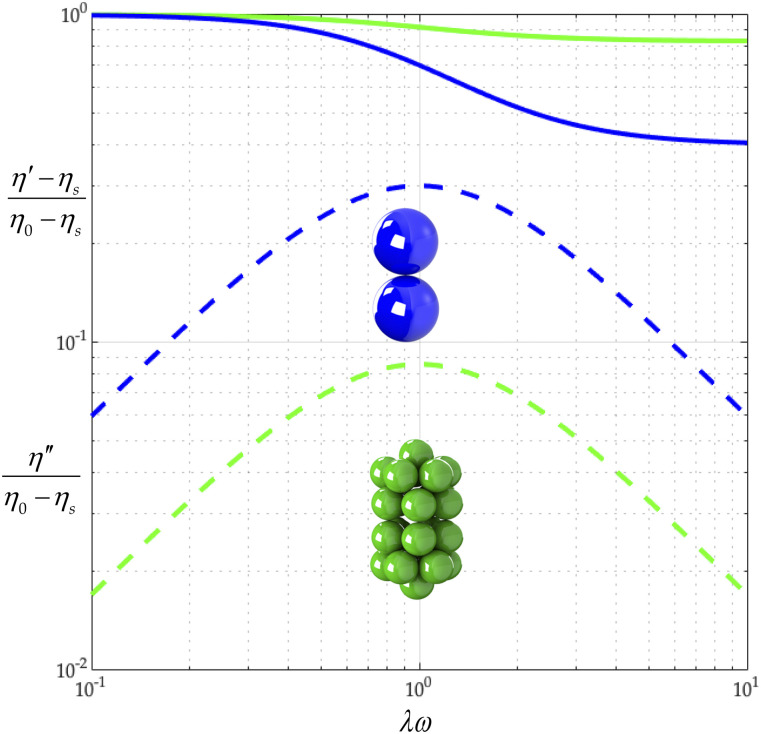
Gemini virus complex viscosity comparison: two osculating beads (green) and twin icosahedra (blue). The solid curve describes (*η*′ − *η*_*s*_)/(*η*_0_ − *η*_*s*_), and the dashed one describes *η*″/(*η*_0_ − *η*_*s*_).

By comparing the values of *λ*_0_*D*_*r*_ in Table VII, we discover that a twin icosahedral gemini model gives a lower rotational diffusivity than for twin osculating beads. Using the values of 2*b*/*aν* for the gemini virus in Table VII, with Eqs. (34) and (35), we construct Fig. 7 from which we first learn that for twin truncated icosahedra, (*η*′ − *η*_*s*_)/(*η*_0_ − *η*_*s*_) descends less sharply than for twin osculating beads. We also learn that for the twin truncated icosahedral structure, $\eta^{\prime \prime }$/(*η*_0_ − *η*_*s*_) falls below that of the twin osculating beads. We thus find that the fine structure of the gemini virus matters, raising viscosity and lowering elasticity.

## VI. ADENOVIRUS

To arrive at the rotational diffusivity of the adenovirus, we follow the method of Sec. II. The adenovirus capsid (not including peplomers) is made up of 252 capsomers in an icosahedral arrangement (plate II of Ref. 58). Twelve of these are “pentons” with five nearest neighbors, and the other 240 are “hexons” with six nearest neighbors.

An icosahedron has 20 triangular faces, 30 edges, and 12 vertices. Each vertex holds a penton, shared by five edges and five faces. The faces are made of two nested triangular arrangements, the inner triangle being made of six hexons. Each of 120 edge hexons is shared by two faces. Each of the 120 face hexons belongs to a single face, with all six of its neighbors lying in that face.

The edges are taken to have length 5*L*, with the interparticle distance *L* equal to the diameter *d* of the capsomer. The 12 vertices sit on the edges of three rectangles with Cartesian coordinates given by cyclic permutations of (±*β*, ±1, 0) scaled by 5*L*/2, where $\beta =\left(1+\sqrt{5}\right)/2$ is the golden ratio. This exploits the fact that $\sqrt{1+\beta^{2}+{\left(\beta -1\right)}^{2}}=2$. We designate the position vectors **V**_*i*_ of the 12 vertices in Table IV. The edge vectors are thus **E**_*ij*_ = **V**_*j*_ − **V**_*i*_, where, for example, **E**_0111_ = **V**_11_–**V**_01_.

**TABLE IV.** Twelve vertices of the adenovirus capsid.

Macromolecule	**V**_*i*_
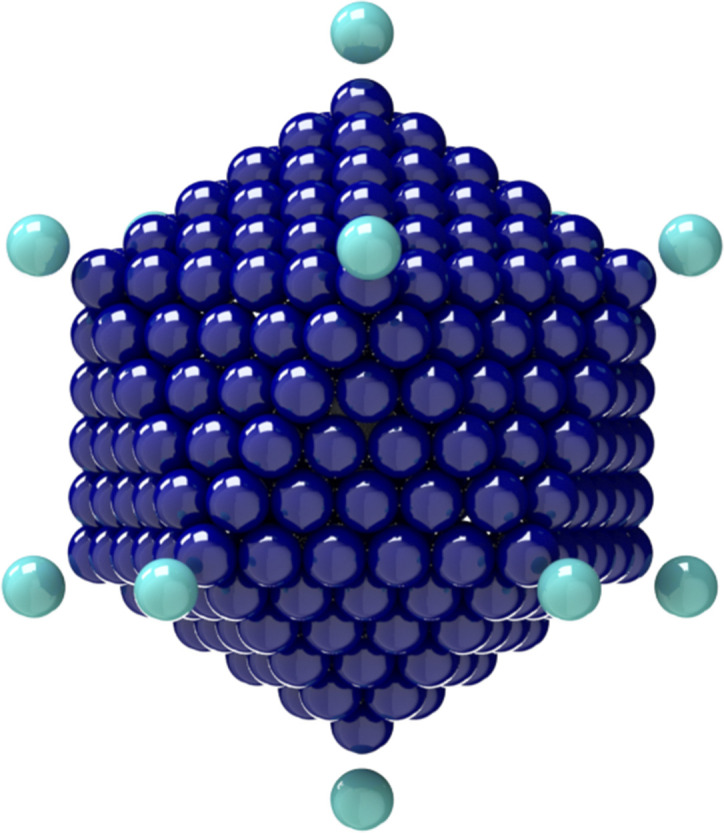	$\begin{array}{c} V_{01}=\frac{5L}{2}\left(\beta ,1,0\right),V_{02}=\frac{5L}{2}\left(\beta ,-1,0\right)\\ V_{03}=\frac{5L}{2}\left(-\beta ,1,0\right),V_{04}=\frac{5L}{2}\left(-\beta ,-1,0\right)\\ V_{05}=\frac{5L}{2}\left(0,\beta ,1\right),V_{06}=\frac{5L}{2}\left(0,\beta ,-1\right)\\ V_{07}=\frac{5L}{2}\left(0,-\beta ,1\right),V_{08}=\frac{5L}{2}\left(0,-\beta ,-1\right)\\ V_{09}=\frac{5L}{2}\left(1,0,\beta \right),V_{10}=\frac{5L}{2}\left(-1,0,\beta \right)\\ V_{11}=\frac{5L}{2}\left(1,0,-\beta \right),V_{12}=\frac{5L}{2}\left(-1,0,-\beta \right) \end{array}$

To describe face beads, we need twelve sets of five triplets *ijk* arising from a counterclockwise enumeration of the five neighboring vertices associated with one vertex. We encode this as follows: The notation 01: 02, 11, 06, 05, 09 means faces *ijk* = 010 211, 011 106, 010 605, 010 509, 010 902. Table V lists our twelve sets.

**TABLE V.** Twelve sets of five triplets describing the faces of the adenovirus capsid.

Vertex *i*	Neighboring vertices *jk*
01	02, 11, 06, 05, 09
02	11, 01, 09, 07, 08
03	04, 10, 05, 06, 12
04	03, 12, 08, 07, 10
05	06, 03, 10, 09, 01
06	05, 01, 11, 12, 03
07	08, 02, 09, 10, 04
08	07, 04, 12, 11, 02
09	10, 07, 02, 01, 05
10	09, 05, 03, 04, 07
11	12, 06, 01, 02, 08
12	11, 08, 04, 03, 06

We associate two edge beads on five edges and two face beads (this is a chiral choice) on five faces with each vertex, so that each of the 12 vertices **V**_*i*_ with *i* = 1, 2, …, 12 is associated with five triplets *ijk* and the following 21 points: the vertex itself, **V**_*i*_, to which we add vectors for the ten edge beads, $\frac{1}{5}E_{ij}$ and $\frac{2}{5}E_{ij}$, and ten face beads, $\frac{1}{5}\left(E_{ij}+E_{ik}\right)$ and $\frac{1}{5}\left(2E_{ij}+E_{ik}\right)$. Following this method, we arrive at the position vectors **R**_*i*_ of the adenovirus capsid beads so that *i* = 1, 2, …, 252.

Oliver, who measured the translational diffusivity of the adenovirus,^37^ overlooked the identity [Eq. (28)] when he wrote “the rotational diffusivity of the adenovirus appears to be zero.” Perhaps this explains why the transport property, rotational diffusivity, has been largely overlooked in virology.

From general rigid bead–rod theory [Eq. (23)], for the characteristic time of the adenovirus, we get

$$\lambda_{0}\equiv \frac{\nu }{72D_{r}},$$ (60)

and then, inserting Eq. (26) gives

$$\lambda_{0}\equiv \frac{L^{2}}{6D_{\text{rot}}}$$ (61)

into which we insert the identity Eq. (27) to get

$$\frac{\lambda_{0}}{L^{2}}\equiv \frac{1}{24D_{tr}}$$ (62)

into which we next insert Eq. (16),

$$\frac{\lambda_{0}}{L^{2}}=\frac{\zeta }{12kT}=\frac{\pi d\eta_{s}}{4kT}=\frac{1}{24D_{tr}}.$$ (63)

Now, from Ref. 37, we have the measured value of the translational diffusivity for the adenovirus at body temperature,

$$D_{tr}≃0.367\times 10^{-7}\text{ c}{\text{m}}^{2}/\text{s}.$$ (64)

Substituting this into Eq. (63),

$$\frac{\lambda_{0}}{L^{2}}=\frac{1}{24\left(0.367\times 10^{-7}\text{ c}{\text{m}}^{2}/\text{s}\right)}=1.14\times 10^{6}\text{ s}/\text{c}{\text{m}}^{2},$$ (65)

which establishes the correspondence between our general rigid bead–rod model of the adenovirus (see in Table VIII) and the adenovirus particle itself.

From the available microscopy (see Fig. 11 of Ref. 59), *r*_*c*_ ≃ 116 nm (between opposing vertices), and the range for the virus radius, made dimensionless with the capsid radius, is given by

$$\frac{6}{5}\leq \frac{r_{v}}{r_{c}}\leq \frac{4}{3}.$$ (66)

We thus position one bead for each adenovirus spike along each of the 12 vertices using *r*_v_/*r*_*c*_ = 5/4 (see Fig. 3), which satisfies Eq. (66). Using *r*_*c*_ ≃ 116 nm and mindful of the adenovirus geometry (see Fig. 3) and its dimensions (see Fig. 4), we get *L* = 12.4 nm. Inserting this into Eq. (65) gives

$$\lambda_{0}=1.75\times 10^{-6}\text{ s,}$$ (67)

namely, the characteristic time, from general rigid bead–rod theory, for the adenovirus.

## VII. CORONAVIRUS

**FIG. 8.**
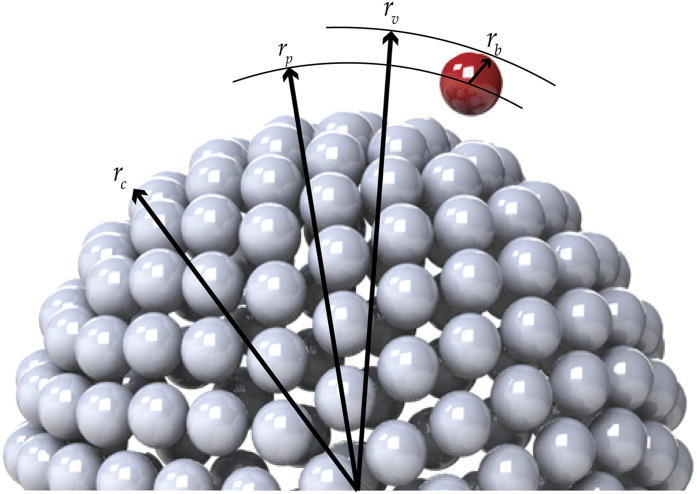
Connections between coronavirus particle dimensions and its general rigid bead–rod model. For the peplomer bulb, we have bead radius $r_{b}\equiv \frac{1}{2}d$ so that for the peplomer height, we have $r_{v}-r_{c}=r_{p}+r_{b}=r_{p}+\frac{1}{2}d$ (see Table X). The *peplomer head radial position* is thus the center to center distance between the peplomer head and the capsid (*r*_v_ − *r*_*b*_).

The coronavirus particle is a biological material whose dimensions are thus known to within biological experimental error. From the available microscopy, *r*_*c*_ ≃ 100 nm–133 nm,^60^ and thus, the range for the virus radius, made dimensionless with the capsid radius, is given by (see Table X and Fig. 8)

$$\frac{5}{4}\leq \frac{r_{v}}{r_{c}}\leq \frac{4}{3}.$$ (68)

Each trimeric peplomer head, consisting of three glycoproteins, is equilateral triangular when viewed along the spike axis (see Fig. 14). For the purposes of the general rigid bead–rod theory, we must replace this trimer with a sphere of radius *r*_*b*_. For this sphere, we choose a diameter, 2*r*_*b*_, matching the length of the equilateral triangle (compare Fig. 8 with Fig. 14). From the available published SARS-CoV spike structure, *r*_*b*_ ≃ 6.5 nm and the peplomer height *r*_v_ − *r*_*c*_ ≃ 13.0 nm.^61^ From this, we learn that the SARS-CoV spike is equidimensional, that is, 2*r*_*b*_/(*r*_v_ − *r*_*c*_) ≃ 1. In general rigid bead–rod theory, we approximate the bulbous SARS-CoV-2 triglycoprotein head with a single bead so that 2*r*_*b*_ = *d*. From the available microscopy, we can see that the range for the triglycoprotein head diameter, made dimensionless with the capsid diameter, is given by (see Table X and Fig. 8)

$$\frac{35}{2}\leq \frac{r_{c}}{r_{b}}\leq \frac{41}{2}.$$ (69)

When viewed through the lens of general rigid bead–rod theory, we learn that the rotational diffusivity of the coronavirus and its associated rheological properties are conferred by the particle shape and not by the ratio *r*_*c*_/*r*_*b*_.

General rigid bead–rod theory requires the particle to be modeled with beads of the same size. For the coronavirus, we match this bead size, *r*_*b*_, to the finest relevant part of the coronavirus structure: the peplomer head (see Fig. 8). The much larger capsid must therefore be beaded, with beads of radii *r*_*b*_.

In this work, we choose the measured peplomer population *N*_*p*_ = 74 (Table X) over the postulated value *N*_*p*_ = 90 (Table X). To construct the specific coronavirus example of Fig. 5 (*N*_*c*_ = 256, *N*_*p*_ = 74, and *r*_v_/*r*_*c*_ = 5/4), we begin by beading a unit sphere for the capsid around which we arrange a constellation of peplomer heads. We get position vectors for these beads by multiplying the 74 point-charge solution to the Thomson problem extracted from Ref. 29 of Ref. 25 by 5/4.

Since the trimeric peplomer heads are charged identically, we expect the spikes to arrange themselves following the polyhedral solutions to the Thomson problem.^25,26^ We learn that these polyhedral solutions are all at least nearly axisymmetric,^31^ but few are exactly so. By *nearly axisymmetric*, we mean that the moments of inertia about the transverse molecular axes, *I*_1_ and *I*_2_, hardly differ. In other words, *nearly axisymmetric* means that the average value of the moments of inertia about the transverse molecular axes, $\frac{1}{2}\left(I_{1}+I_{2}\right)$, hardly differs from *I*_1_. In dimensionless terms, nearly axisymmetric thus means

$$\left|\frac{I_{2}-I_{1}}{2I_{1}}\right|\ll 1$$ (70)

to which Eqs. (34) and (35) are subject. For the reported polyhedral solutions to the Thomson problem,^25,26^

$$\left|\frac{I_{2}-I_{1}}{2I_{1}}\right|=o\left(10^{-4}\right),$$ (71)

which satisfies Eq. (70). The method of this section can be used for any spiked virus with a spherical capsid, including the insect Pariacoto virus [Fig. 22(a) of Ref. 62].

**FIG. 9.**
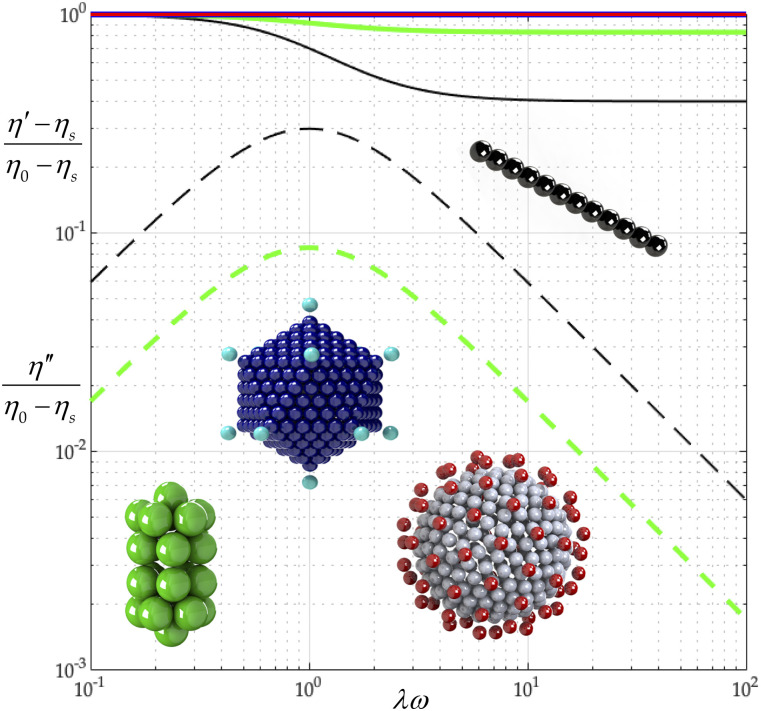
Tobacco mosaic (black), gemini (green), adeno (blue), and corona (red) complex viscosity comparison. The solid curve describes (*η*′ − *η*_*s*_)/(*η*_0_ − *η*_*s*_), and the dashed one describes *η*″/(*η*_0_ − *η*_*s*_).

**FIG. 10.**
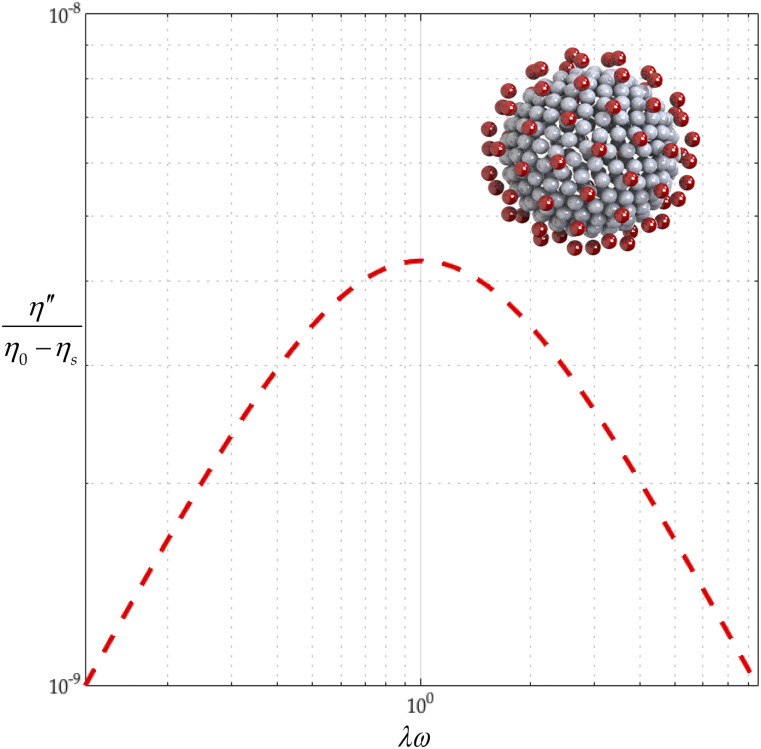
Corona (red) elastic complex viscosity, *η*″/(*η*_0_ − *η*_*s*_), curve.

Figure 9 combines results on all four of our viruses (from Sec. IV to the present section) using the calculated 2*b*/*aν* values from Tables VI–IX (row 6 of Table IX). From Fig. 9, we learn that (*η*′ − *η*_*s*_)/(*η*_0_ − *η*_*s*_) curves for the adeno- and coronavirus bead–rod models descend less sharply than those of tobacco mosaic or gemini viruses. From Fig. 10, we learn that the higher the complexity (coronavirus), the lower the *η*″/(*η*_0_ − *η*_*s*_), and thus, the lower the dimensionless elasticity. We also learn that adenovirus is *spherically symmetrical* (*I*_1_ = *I*_2_ = *I*_3_), and thus, it is not associated with $\eta^{\prime \prime }$/(*η*_0_ − *η*_*s*_).

**TABLE VI.** Tobacco mosaic characteristics from general rigid bead–rod theory.

Macromolecule	$\frac{I_{1}}{mL^{2}}$, $\frac{I_{2}}{mL^{2}}$	$\frac{I_{3}}{mL^{2}}$	*a*	*b*	*ν*	$\frac{2b}{a\nu }$	$\frac{\eta_{0}-\eta_{s}}{nkT\lambda }$	$\frac{\lambda }{\lambda_{0}}$	*λ*_0_*D*_*r*_	$\frac{\Psi_{0,1}}{\lambda \left(\eta_{0}-\eta_{s}\right)}$
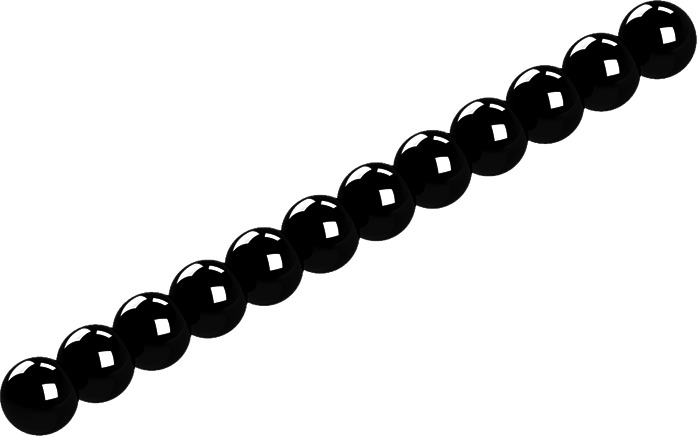	143	0	$\frac{286}{15}$	$\frac{3}{5}$	$\frac{6}{143}$	$\frac{3}{2}$	1	286	$\frac{1}{1716}$	$\frac{6}{5}$

**TABLE VII.** Gemini characteristics from general rigid bead–rod theory: Twin icosahedra vs two osculating beads models.

Macromolecule	$\frac{I_{1}}{mL^{2}}$, $\frac{I_{2}}{mL^{2}}$	$\frac{I_{3}}{mL^{2}}$	*a*	*b*	*ν*	$\frac{2b}{a\nu }$	$\frac{\eta_{0}-\eta_{s}}{nkT\lambda }$	$\frac{\lambda }{\lambda_{0}}$	*λ*_0_*D*_*r*_	$\frac{\Psi_{0,1}}{\lambda \left(\eta_{0}-\eta_{s}\right)}$
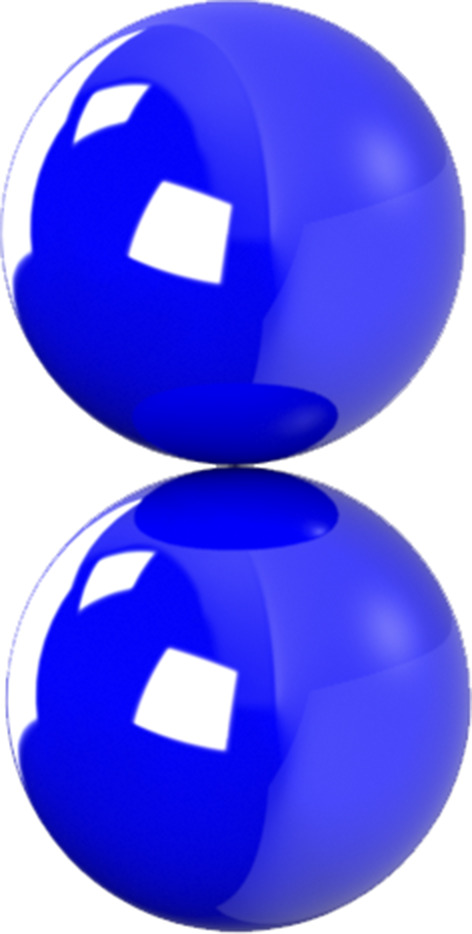	$\frac{1}{2}$	0	$\frac{1}{15}$	$\frac{3}{5}$	12	$\frac{3}{2}$	1	1	$\frac{1}{6}$	$\frac{6}{5}$
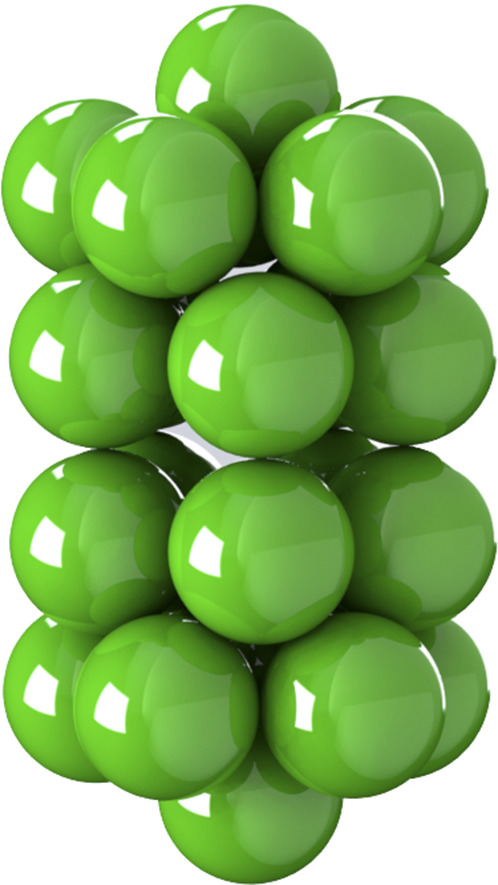	35.02	14.47	11.67	0.2065	0.1713	0.2065	1.207	70.04	0.002 379	0.3423

**TABLE VIII.** Adenovirus characteristics from general rigid bead–rod theory.

Macromolecule	$\frac{I_{1}}{mL^{2}}$, $\frac{I_{2}}{mL^{2}}$	$\frac{I_{3}}{mL^{2}}$	*a*	*b*	*ν*	$\frac{2b}{a\nu }$	$\frac{\eta_{0}-\eta_{s}}{nkT\lambda }$	$\frac{\lambda }{\lambda_{0}}$	*λ*_0_*D*_*r*_	$\frac{\Psi_{0,1}}{\lambda \left(\eta_{0}-\eta_{s}\right)}$
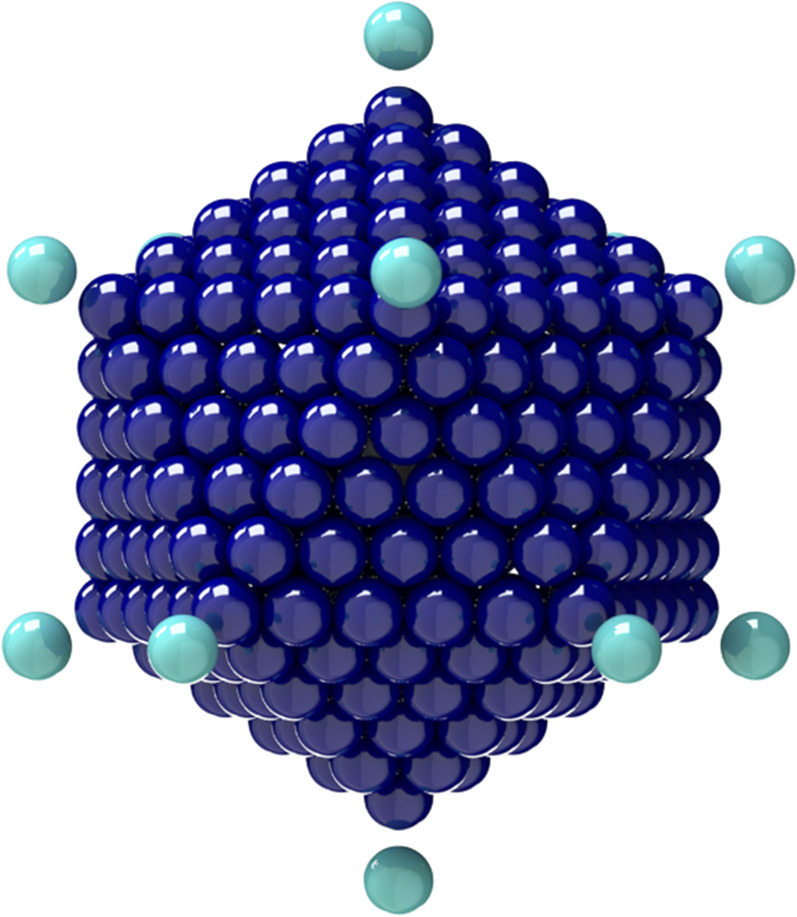	3082	3082	1541	0	0.001 947	0	$\frac{3}{2}$	6163	0.000 027 04	0

**TABLE IX.** Coronavirus characteristics for different capsid beadings from general rigid bead–rod theory.

SARS-CoV-2	$\frac{I_{1}}{mL^{2}}$	$\frac{I_{2}}{mL^{2}}$	$\frac{I_{3}}{mL^{2}}$	*a*	*b*	*ν*	$\frac{2b}{a\nu }$	$\frac{\eta_{0}-\eta_{s}}{nkT\lambda }$	$\frac{\lambda }{\lambda_{0}}$	*λ*_0_*D*_*r*_	$\frac{\Psi_{0,1}}{\lambda \left(\eta_{0}-\eta_{s}\right)}$
*N*_*c*_ = 16, *N*_*p*_ = 74	87.76	87.71	87.79	43.88	9.52 × 10^−8^	6.84 × 10^−2^	6.34 × 10^−8^	1.50	175.51	9.50 × 10^−4^	1.27 × 10^−7^
*N*_*c*_ = 32, *N*_*p*_ = 74	98.42	98.37	98.46	49.22	7.57 × 10^−8^	6.10 × 10^−2^	5.04 × 10^−8^	1.50	196.85	8.47 × 10^−4^	1.01 × 10^−7^
*N*_*c*_ = 64, *N*_*p*_ = 74	143.77	143.66	143.80	71.90	1.86 × 10^−8^	4.17 × 10^−2^	1.24 × 10^−8^	1.50	287.55	5.80 × 10^−4^	2.48 × 10^−8^
*N*_*c*_ = 128, *N*_*p*_ = 74	162.41	162.40	162.45	81.21	3.28 × 10^−8^	3.69 × 10^−2^	2.18 × 10^−8^	1.50	324.82	5.13 × 10^−4^	4.36 × 10^−8^
*N*_*c*_ = 256, *N*_*p*_ = 74	247.76	247.70	247.79	123.88	1.19 × 10^−8^	2.24 × 10^−2^	7.96 × 10^−9^	1.50	495.51	3.36 × 10^−4^	1.60 × 10^−8^
*N*_*c*_ = 510, *N*_*p*_ = 74	417.10	417.02	417.12	208.56	7.50 × 10^−10^	1.44 × 10^−2^	4.50 × 10^−10^	1.50	834.21	2.00 × 10^−4^	9.0 × 10^−10^

### A. Capsid beading

In this subsection, we vary the capsid beading of the coronavirus particle, *N*_*c*_, and fix the peplomer bead number, *N*_*p*_ = 74. We model the different capsid beadings, *N*_*c*_ = 16, 32, 64, 128, 256, 510, and thus construct Table IX. By comparing the values of *λ*/*λ*_0_ in Table IX, we learn that making the capsid beading finer (increasing *N*_*c*_) increases the relaxation time. By examining the values of (*η*_0_ − *η*_*s*_)/*nkTλ* in Table IX, we also learn that increasing *N*_*c*_ does not affect the polymer contribution to zero-shear viscosity, since (*η*_0_ − *η*_*s*_)/*nkTλ* remains 1.5. From Table IX, we learn that dimensionless rotational diffusivity, *λ*_0_*D*_*r*_, decreases with *N*_*c*_.

**FIG. 11.**
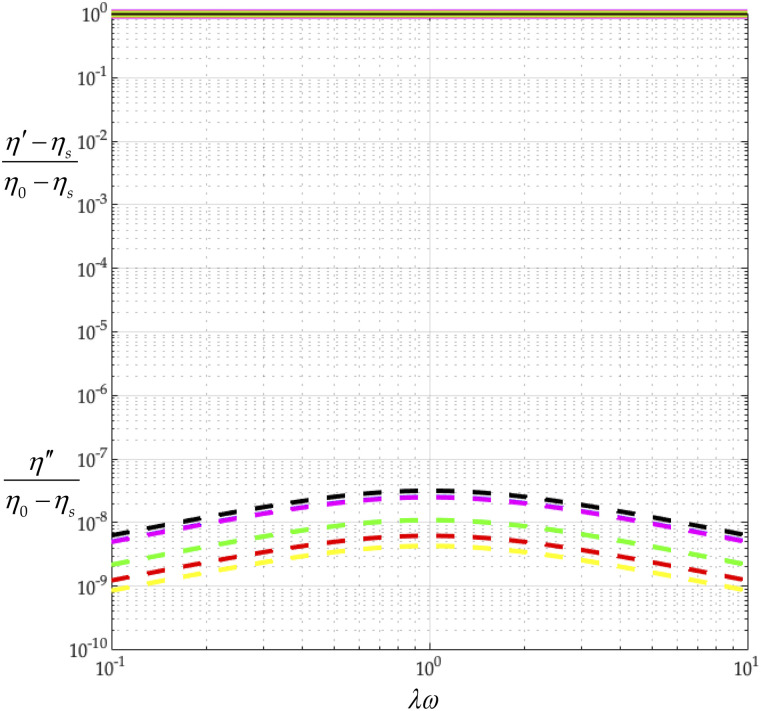
Effect of capsid osculated beadings on complex viscosity (*N*_*c*_ = 16, 32, 64, 128, 256, 510, *N*_*p*_ = 74, *r*_v_/*r*_*c*_ = 5/4, and *L* = *d*). *N*_*c*_ = 16 (black), *N*_*c*_ = 32 (blue), *N*_*c*_ = 64 (red), *N*_*c*_ = 128 (green), *N*_*c*_ = 256 (yellow), and *N*_*c*_ = 510 (magenta). The solid curve describes (*η*′ − *η*_*s*_)/(*η*_0_ − *η*_*s*_), and the dashed one describes *η*″/(*η*_0_ − *η*_*s*_).

Using the values of 2*b*/*aν* for all coronavirus capsid beadings in Table VII, with Eqs. (34) and (35), we construct Fig. 11 from which we first learn that increasing *N*_*c*_ does not change (*η*′ − *η*_*s*_)/(*η*_0_ − *η*_*s*_). A coronavirus model thus always gives a nearly constant viscosity (nearly spherically symmetric).

### B. Peplomer population

**FIG. 12.**
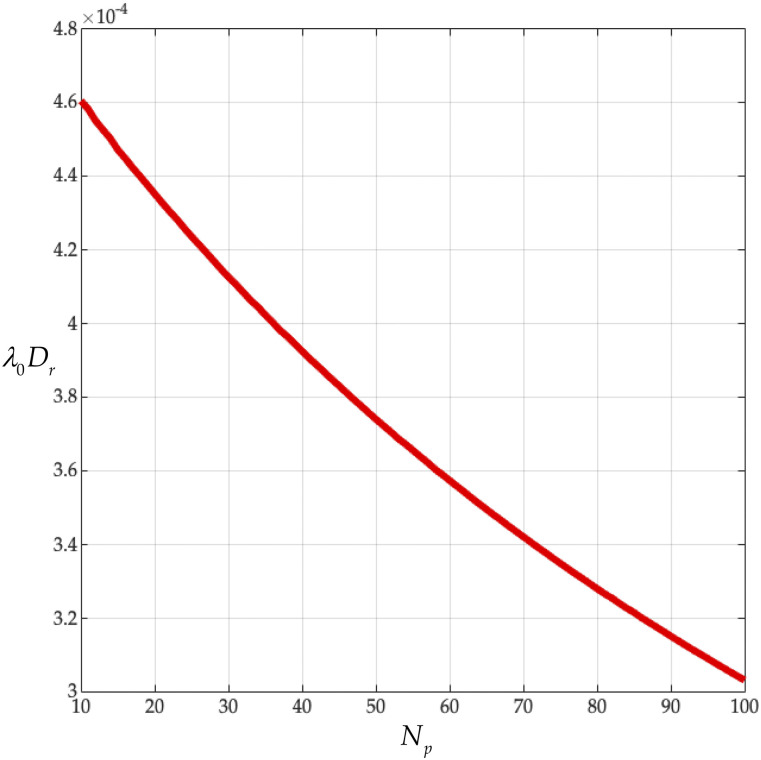
Dimensionless rotational diffusivity *λ*_0_*D*_*r*_ from Eq. (23) vs peplomer population *N*_*p*_ (*N*_*c*_ = 256).

**FIG. 13.**
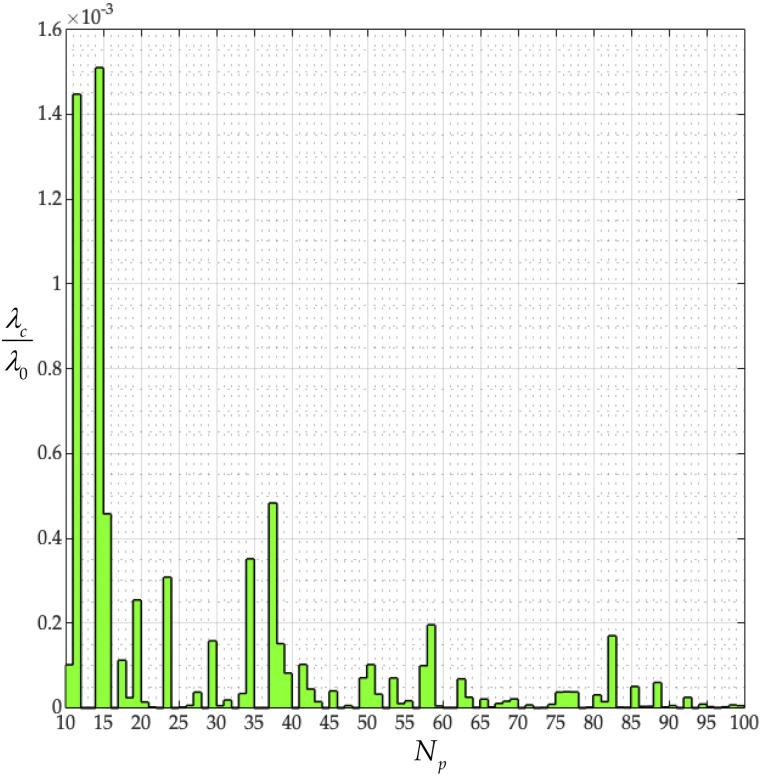
Dimensionless structure-dependent characteristic time, *λ*_*c*_/*λ*_0_, vs peplomer population, *N*_*p*_ (*N*_*c*_ = 256), from Eq. (40).

In this subsection, we fix the capsid beading of the coronavirus particle at *N*_*c*_ = 256 and vary the peplomer bead population over 10 ≤ *N*_*p*_ ≤ 100 to get Figs. 12 and 13. From Fig. 12, we learn that rotational diffusivities of the coronavirus, made dimensionless with the constant *λ*_0_, are of order 10^−4^. Specifically, for the measured peplomer population, *N*_*p*_ = 74 (see Table X), we get *λ*_0_*D*_*r*_ = 3.36 × 10^−4^. This value exceeds the dimensionless diffusivity of the adenovirus (Table VIII) and falls below those of the tobacco mosaic (Table VI) and gemini viruses (Table VII). The binding interval for the SARS-CoV-2 particle exceeds 3 min [see Fig. 3(A) of Ref. 1 and Refs. 61 and 63], and thus, it would appear that coronavirus peplomer binding prefers *λ*_0_*D*_*r*_ = *o*(10^−4^).

**TABLE X.** Physical dimensions of coronaviruses (see Fig. 8). *p* ≡ postulated.

Macromolecule	*r*_*c*_ (nm)	*r*_v_ (nm)	$\frac{r_{v}}{r_{c}}$	*r*_v_ − *r*_*c*_ (nm)	*r*_*b*_ (nm)	*N*_*p*_	References
SARS-CoV-2		30–70		9–12			64
SARS-CoV		39					68
SARS-CoV		44–47					Section 3 of Ref. 60
SARS-CoV						74, 90^*p*^	Section 4.1 of Ref. 60
SARS-CoV	100–133		1.25–1.53				Figure 1(D) of Ref. 60
SARS-CoV				13.0	6.5		6CRZ.PDB from Ref. 61

Equation (39) defines a structure-dependent characteristic time, *λ*_*c*_, and the ratio of the first normal stress coefficient to the viscosity at zero shear rate. Specifically, we next explore how *λ*_*c*_ depends on the peplomer population. Nondimensionalizing *λ*_*c*_ with the constant *λ*_0_, produces the stairstep plot of Fig. 13, which is not monotonic. From Fig. 13, we learn that the characteristic times of the coronavirus particles, made dimensionless with the *λ*_0_, over 10 ≤ *N*_*p*_ ≤ 100, fall below 10^−4^. In other words, the elasticity of the coronavirus particles is slight and is not monotonic with *N*_*p*_. Furthermore, at the measured peplomer population of *N*_*p*_ = 74 (Table X), *λ*_*c*_/*λ*_0_ is vanishingly small.

In this subsection, we have explored the role of *N*_*p*_ on the transport properties of the coronavirus particle and found that its precise value matters. Such precise values for *N*_*p*_, be it for SARS-CoV-2, SARS-CoV, or any other spiked virus with a spherical capsid, have yet to be reported.

## VIII. CONCLUSION

We find that our *ab initio* calculations agree with the observed complex viscosity of the tobacco mosaic virus suspension (Fig. 6). From our analysis of the gemini virus suspension, we learn that the fine detail of the virus structure governs its rotational diffusivity (Fig. 7). We find that combining our *ab initio* calculations with the observed rotational diffusivity of the adeno suspension yields the characteristic time, from general rigid bead–rod theory, for the adenovirus (Sec. VI). Finally, from our analysis of the coronavirus suspension (Sec. VII), we learn that its rotational diffusivity descends monotonically with its peplomer population (Fig. 12).

In Sec. VII, we tackled spiked viruses with spherical capsids for which *b* ≃ 0. However, histologically, SARS-CoV-2 capsids present with pleomorphism (Fig. 3 of Ref. 64). We leave the rotational diffusivity of spiked viruses of non-spherical (including ellipsoidal) capsids, for which *b* > 0, for another day.

In Sec. IV, we learned how to deduce the rotational diffusivity of a virus by fitting the measured values of the real and imaginary parts of the complex viscosity function to the main results from general rigid bead–rod theory [Eqs. (34) and (35)]. However, complex viscosity measurements on SARS-CoV-2 suspensions are unavailable, and their measurement is understandably dangerous. Perhaps the method of microrheology, which requires just one drop of SARS-CoV-2 suspension, will yield its complex viscosity measurements (see Refs. 65 and 66 and Chaps. 4 and 5 of Ref. 67).

If the dimer target is projected onto the peplomer orientation distribution function, the integral (in phase space) under this projection gives the probability of one spike aligning properly for fusion, *p*. Since, for fusion, we need two adjacent spikes to align, the probability falls well below *p*. Equations (1) and (2) consider just one special case of alignment. We leave the integral (in phase space) over all possible alignments for another day.

Our macromolecular bead–rod model viruses are suspended in a Newtonian solvent. We neglect interactions of the solvent velocity fields, be they between nearest beads (see Refs. 48 and 49 and Sec. 14.6 of Ref. 22) or nearest macromolecules. We leave the effects of these hydrodynamic interactions on rotational diffusivity for another day.

**FIG. 14.**
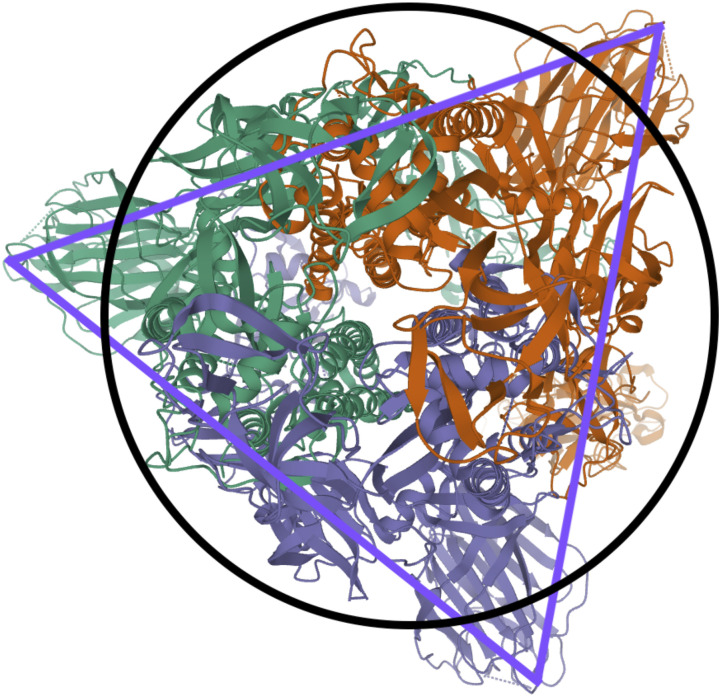
In this work, we replace the peplomer bulk with sphere (a) inscribed in the trimer, thus neglecting its triangularity. Future work shall improve upon this by inscribing the trimer (b) into three osculating beads.^61^

Whereas the bulbous head of a peplomer is trimeric and therefore triangular, in this work, we have represented the bulb with a single bead. Figure 14 illustrates this model. We thus propose incorporating the triangularity of the bulbous peplomer by replacing its head with three identically charged osculating beads. The potential energy minimization for these three-beaded bulbs will, of course, produce new and interesting polyhedra differing from the Thomson solutions used herein.^25,26^ We leave this potential energy minimization, polyhedra discovery, and corresponding *ab initio* rotational diffusivity calculation for another day.

Under the microscope, we see some agglomeration of coronavirus particles, mechanically interlocked by interdigitation of the bulbous spikes [see Fig. 1(D) of Ref. 60]. The simplest of these agglomerates is a pair. We leave the calculation of the diffusivity of such interdigitated coronavirus structures from general rigid bead–rod theory for another day.

For this work, for both adenovirus and coronavirus, we chose the ratio *r*_v_/*r*_*c*_ = 5/4 (Figs. 3 and 5, respectively), which is consistent with the available microscopy [Eqs. (66) and (68), respectively]. We leave the exploration of the rotational diffusivities over these dimensionless spike length ranges for another day.

Whereas in engineering, the complex viscosity function has a broad diversity of applications including polymer or suspension processing, for virus suspensions, its main use is for determining rotational diffusivity. The uninitiated might expect that our complex viscosity equations for the adenovirus [Eqs. (34) and (35) with Table VIII] or coronavirus [Eqs. (34) and (35) with Table IX] suspensions might be useful for cough or sneeze cloud modeling. However, such cloud droplets are not merely virus particle suspensions but suspend the materials that virus infected lungs or nasal passages produce.

More broadly, our vision here is that a handbook of general rigid bead–rod virus models be generated eventually from which the transport properties of any included virus might be calculated and then compared. Such a handbook might thus lead us to deepen our understanding of the relation between the rotational diffusivities of virus particles and their intricate shapes.

## DATA AVAILABILITY

The data that support the findings of this study are available within the article.
